# Colonization, spread and persistence of *Salmonella* (Typhimurium, Infantis and Reading) in internal organs of broilers

**DOI:** 10.1016/j.psj.2024.103806

**Published:** 2024-05-01

**Authors:** Jinquan Wang, Davis A. Fenster, Sasikala Vaddu, Sujitha Bhumanapalli, Jasmine Kataria, Gaganpreet Sidhu, Cortney Leone, Manpreet Singh, Rami A. Dalloul, Harshavardhan Thippareddi

**Affiliations:** ⁎ Department of Poultry Science, University of Georgia, Athens, GA 30602, USA; † Department of Food Science and Technology, University of Georgia, Athens, GA 30602, USA

**Keywords:** broiler, *Salmonella*, translocation, persistence, serovar

## Abstract

Transfer of *Salmonella* to internal organs of broilers over a 35 d grow-out period was evaluated. A total of 360 one-day old chicks were placed in 18 floor pens of 3 groups with 6 replicate pens each. On d 0, broilers were orally challenged with a cocktail of *Salmonella* (equal population of marked serovars; nalidixic acid-resistant *S*. Typhimurium, rifampicin-resistant *S*. Infantis, and kanamycin-resistant *S*. Reading) to have 3 groups: L (low; ∼2 log CFU/bird); M (medium; ∼5 log CFU/bird); and H (High; ∼8 log CFU/bird). On d 2, 7 and 35, 4 birds/pen were euthanized and ceca, liver, and spleen samples were collected aseptically. Gizzard samples (4/pen) were collected on d 35. The concentration of *Salmonella* in liver and spleen were transformed to binary outcomes (positive and negative) and fitted in *glm* function of R using cecal *Salmonella* concentrations (log CFU/g) and inoculation doses (L, M, and H) as inputs. On d 2, H group showed greater (*P* ≤ 0.05) cecal colonization of all 3 serovars compared to L and M groups. However, M group showed greater (*P* ≤ 0.05) colonization of all 3 serovars in the liver and spleen compared to L group. *Salmonella* colonization increased linearly in the ceca and quadratically in the liver and spleen with increasing challenge dose (*P* ≤ 0.05). On d 35, L group had greater (*P* ≤ 0.05) *S*. Infantis colonization in the ceca and liver compared to M and H groups (*P* ≤ 0.05). Moreover, within each group on d 35, the concentration of *S*. Reading was greater than those of *S*. Typhimurium and *S*. Infantis for all 3 doses in the ceca and high dose in the liver and gizzard (*P* ≤ 0.05). *Salmonella* colonization diminished in the ceca, liver, and spleen during grow-out from d 0 to d 35 (*P* ≤ 0.05). On d 35, birds challenged with different doses of *Salmonella* cocktail showed a similar total *Salmonella* spp. population in the ceca (ca. 3.14 log CFU/g), liver (ca. 0.54 log CFU/g), spleen (ca. 0.31 log CFU/g), and gizzard (ca. 0.42 log CFU/g). Estimates from the fitted logistic model showed that one log CFU/g increase in cecal *Salmonella* concentration will result in an increase in relative risk of liver and spleen being *Salmonella*-positive by 4.02 and 3.40 times (*P* ≤ 0.01), respectively. Broilers from H or M group had a lower risk (28 and 23%) of being *Salmonella*-positive in the liver compared to the L group when the cecal *Salmonella* concentration is the same (*P* ≤ 0.05). Oral challenge of broilers with *Salmonella* spp. with various doses resulted in linear or quadratic increases in *Salmonella* colonization in the internal organs during early age and these populations decreased during grow-out (d 35). This research can provide guidance on practices to effectively mitigate the risk of *Salmonella* from chicken parts and enhance public health.

## INTRODUCTION

Salmonellosis is a major foodborne illness and has been linked to consumption of poultry products including meat and eggs (Antunes et al., 2016). Interagency Food Safety Analytics Collaboration (**IFSAC**) estimated over 23% of salmonellosis can be contributed to consumption of chicken and turkey products (IFSAC, 2021). Adoption of strategies at preharvest to reduce both *Salmonella* prevalence and concentration is required to achieve a greater reduction in *Salmonella* loads in broiler meat.

Among all serovars, *Salmonella* Typhimurium and *S.* Enteritidis were the most studied serovars in broilers as they are responsible for most human cases (Finstad et al., 2012; Lamas et al., 2018, Punchihewage-Don et al., 2022). *S*. Infantis is an emerging serovar that is increasingly causing greater human illness in the U.S. and Europe (Alba et al., 2020; Tack et al., 2020). The incidence of *S*. Infantis has increased since 2016 and accounted for 17.7% (30/170) of *Salmonella*-positive samples in 2020 in the U.S. (Siceloff et al., 2022). The European Food Safety Authority (EFSA, 2018) identified *S*. Infantis as the most frequent serovar in broiler flocks (45.6%) and broiler meat (50.6%) and showed an increasing prevalence in breeder flocks. *S*. Reading is rarely associated with human illness from chicken products. Recent outbreaks linked to consumption of turkey products have made *S*. Reading a prominent serovar for public health (Hassan et al., 2019). Few studies have evaluated colonization and translocation of *S*. Infantis and *S*. Reading to internal organs during grow-out of broilers.

*Salmonella* challenge models (seeder birds or oral/cloacal gavage) are often used to induce gastrointestinal (**GI**) tract colonization to evaluate the efficacy of dietary interventions on mitigating *Salmonella* colonization in broilers. In such studies, chicks are commonly challenged with *Salmonella* at a high dose of 6 to 8 log CFU/mL through the oral route to ensure a successful colonization, and cecal *Salmonella* concentration was enumerated through early stages of the birds’ life to evaluate the efficacy of tested product (Shanmugasundaram et al., 2019; Yadav et al., 2022). Regardless of the differences in challenge models used in broiler research, limited literature on the effect of challenge dose on colonization of *Salmonella* in the GI tract and translocation to other internal parts is available, particularly simulating commercial production practices such as raising birds in floor pens.

The majority of studies on *Salmonella* spread to broiler parts reported prevalence as a main indicator of movement and persistence in liver or spleen (Adhikari et al., 2020; Choi et al., 2022). However, reporting *Salmonella* prevalence provides limited information on colonization and translocation to internal organs as the food safety risk of *Salmonella* in those products is potentially related to their concentrations, and risk mitigation through preparation and cooking practices (Harris et al., 2003). Reports on *Salmonella* concentration in broiler edible organs such as liver and gizzard especially at the preharvest stage are limited, and this information can be valuable to develop risk mitigation recommendations for consumers of these products. The objective of this study was to quantify the transfer and persistence of *Salmonella* spp. to various internal organs of broilers that were orally inoculated with different doses of a *Salmonella* cocktail containing the serovars Typhimurium, Infantis and Reading.

## MATERIALS AND METHODS

The experimental protocol was reviewed and approved by the University of Georgia Institutional Animal Care and Use Committee.

### *Salmonella* Challenge and Birds Husbandry

Three *Salmonella* serovars, nalidixic acid-resistant *S*. Typhimurium (**ST**) obtained from the U.S. National Poultry Research Center (NPRC-USDA, Athens, GA), rifampicin-resistant strain of *S*. Infantis (5 isolates; SI) and kanamycin-resistant *S*. Reading (5 isolates; SR) obtained from the Food Safety and Inspection service (**FSIS**) were used in this study. Cultures of the bacteria were maintained as frozen stocks. The *Salmonella* serovar cocktail for the challenge was prepared as previously described (Yadav et al., 2022). Briefly, 1 d prior to challenge, 5 colonies of ST isolated from BG Sulfa agar (BGS; Difco**,** Sparks, MD) containing 200 ppm nalidixic acid were used to inoculate 10 mL of tryptic soy broth (TSB; Remel, Lenexa, KS) also supplemented with 200 ppm nalidixic acid, the cultures were then incubated for 18 to 24 h at 35 ± 1°C. Preparation of SI and SR inoculum followed similar procedure except for the corresponding antibiotic marker (rifampicin and kanamycin) supplemented to BGS and TSB. Five tubes of each strain were pooled into three 50 mL conical tubes and centrifuged at 7,838 × *g* for 10 min at 4°C (Model 5430R, Eppendorf North America, Enfield, CT) to collect the cells. The individual pellets of each serovar were re-suspended with 10 mL 0.1% buffered peptone water (BPW; Difco TM, Sparks, MD) and centrifuged again. The supernatant was removed to wash away the antibiotics, the pellet re-suspended in 5 mL of BPW, and the OD values of suspension were read at a wavelength 400 nm using spectrometer (Genesys 10S UV-VIS, Thermo Scientific, Waltham, MA). The inoculum was serially diluted using PW to prepare the target inoculum dose: L (∼2 log CFU/mL), M (∼5 log CFU/mL) and H (∼8 log CFU/mL).

A total of 360 Cobb male broilers were obtained from a local hatchery (Cobb Vantress, Cleveland, GA) and randomly allocated to the 3 treatment groups based on the 3 doses of *Salmonella* cocktail inoculum (1 mL/bird containing equal population of marked serovars ST, SI, and SR). Birds were reared in floor pens with fresh litter in an environmentally controlled room at the UGA Poultry Research Center. The room temperature and lighting schedule were set following the Cobb Broiler Management Guide (Cobb-Vantress, 2018b). A corn and soybean meal-based diet meeting or exceeding Cobb Nutrition Guide requirements (Cobb-Vantress, 2018a) was fed to all treatment groups. Birds had *ad libitum* access to water and feed throughout the 35-d grow-out period.

### Sample collection and *Salmonella* Enumeration

On d 2, 7, and 35, internal organs (ceca, liver, and spleen) of 4 birds from each pen were aseptically collected for *Salmonella* enumeration. The cecal sample weights ranged from 0.5 to 1.5 g, 1 to 3 g and 10 to 20 g on d 2, 7, and 35, respectively. Similarly, the liver and spleen samples weighed 1 to 3 g, 4 to 8 g, and 30 to 45 g and 0.1 to 0.3 g, 1 to 3 g and 2 to 4 g, respectively on d 2, 7, and 35 d. Additionally, the gizzard was aseptically everted to expose the inner layer using a scalpel, the contents were removed on d 35 and the gizzard was used for microbiological enumeration. All samples were placed in sterile filter bags (Nasco, Weber Scientific, Hamilton Township, NJ) and weighed.

Chilled BPW (10 mL for samples [ceca, liver, and spleen] from d 2 and 7; 40 mL for samples from d 35) was added into each sample and stomached (Neutec Group Inc., Farmingdale, NY) for 1 min. Chilled BPW (40 mL) was added to each gizzard sample and stomached as described. Serial dilutions were prepared in sterile 0.1% BPW, and appropriate dilutions were plated on duplicate BGS agar plates supplemented with nalidixic acid, rifampicin, or kanamycin to enumerate the ST, SI, and SR, respectively. The plates were incubated for 24 h at 35 ± 1°C, typical colonies were enumerated and reported as log_10_ CFU/g. Simultaneously, an aliquot of the sample (3 mL) was added to BAX MP medium (Hygiena, Camarillo, CA) supplemented with nalidixic acid, rifampicin, or kanamycin (57 mL for ceca sample and 3 mL for liver and spleen samples) and incubated for 24 h at 35 ± 1°C. *Salmonella* prevalence was determined using BAX System SalQuant (Hygiena, Camarillo, CA) for samples below the detection limit.

### Statistical Analysis

All enumeration data were log-transformed and analyzed using one-way ANOVA using the GLM procedure of SAS OnDemand for Academics (SAS Institute, Cary, NC). Separation of means was performed using Tukey's range test at α = 0.05. Polynomial orthogonal contrast was used to determine the linear and quadratic effects of the challenge dose.

Additionally, logistic regression was performed in R (Version 4.0.2,R Core Team, 2021) using *glm* function with *family = binomial* argument. *Salmonella* prevalence was used as the response, and the cecal *Salmonella* concentration (log CFU/g) and treatment (L, M, and H) were used to determine the correlation. The *Salmonella* concentration (log CFU/g) in liver and spleen was transformed into prevalence (positive and negative). Individual birds were used as the data input (n = 3 treatments × 6 replicates × 4 birds × 3 time points = 216 total). Figures were generated as change of probability of liver/spleen being tested positive (0–100%) against cecal *Salmonella* concentration (log CFU/g) using *ggplot2* package.

## RESULTS

### *Colonization* and *Translocation* of *Salmonella in Broiler Internal Organs*

*Salmonella* population in the L, M, and H challenge doses were 2.82, 5.13, and 8.39 log CFU/mL, respectively. *Salmonella* concentration in the ceca increased linearly (*P* ≤ 0.01) with the challenge dose, from 2 to 8 log CFU/bird, 2 d post-inoculation (**dpi**) for all 3 serovars and the pooled count (Table 1). Day-old chicks challenged with all 3 doses were able to establish colonization of *Salmonella* in the ceca with 6.75, 7.47, and 8.50 log CFU/g for L, M, and H groups at 2 dpi, respectively. Pooled *Salmonella* concentration of 3 serovars in the liver and spleen from M dose group birds were higher than L dose group (*P* ≤ 0.05). The concentrations of 3 individual *Salmonella* serovars and their pooled counts in the liver and spleen showed a quadratic increase (*P* ≤ 0.01) as the challenge dose increased.

**Table 1:** *Salmonella* colonization and persistence (log CFU/g) in the ceca and internal organ invasion (liver and spleen) in day-old chicks during grow out as affected by different serovars (Typhimurium, Infantis, and Reading) and challenge dose (approx. 2, 5, and 8 log CFU/chick).

Part	Dose/*p*-values	*S*. Typhimurium	*S*. Infantis	*S*. Reading	Pooled results of 3 serovars
Ceca	Low	5.10 ± 1.22^a^	5.74 ± 0.47^a^	6.04 ± 0.75^a^	6.75 ± 0.68
	Med	6.83 ± 0.27^b^	6.95 ± 0.26^b^	6.90 ± 0.16^b^	7.47 ± 0.13
	High	7.92 ± 0.61^c^	7.82 ± 0.43^c^	7.72 ± 0.33^c^	8.50 ± 0.33
	Linear	<0.001	<0.001	0.0026	<0.001
	Quad	0.7970	0.5074	0.1536	0.1311
Liver	Low	1.93 ± 1.24^a^	1.55 ± 1.41^a^	1.77 ± 1.19^a^	2.79 ± 1.21
	Med	3.87 ± 0.31^b^	3.28 ± 0.37^b^	3.49 ± 0.63^b^	4.34 ± 0.48
	High	2.81 ± 0.8^b^	2.43 ± 0.92^ab^	2.40 ± 0.68^ab^	3.37 ± 0.64
	Linear	0.0706	0.0926	0.1401	0.3637
	Quad	0.0025	0.0157	0.0031	0.0444
Spleen	Low	1.28 ± 0.65^a^	0.79 ± 0.84^a^	1.35 ± 1.06^a^	1.88 ± 1.01
	Med	3.30 ± 0.68^b^	2.97 ± 0.89^b^	3.29 ± 1.06^b^	4.20 ± 0.81
	High	2.42 ± 1.28^ab^	2.28 ± 1.26^b^	2.52 ± 1.33^ab^	3.41 ± 0.95
	Linear	0.048	0.0226	0.1016	0.1722
	Quad	0.0066	0.0125	0.0332	0.0423

Same superscripts (^abc^) within the same column indicate no significant differences (*P* > 0.05); Same superscripts (^xy^) within the first 3 row (3 serovars of *S*. Typhimurium, *S*. Infantis, and *S*. Reading) indicate no significant differences (*P* > 0.05).

The pooled counts of 3 serovars in the ceca, liver, and spleen at 7 dpi increased quadratically (*P* ≤ 0.05) with increasing challenge dose, reaching a peak for Med dose birds (Table 2). Chicks challenged with a L dose of *Salmonella* showed greater SI*^Rif^* colonization (6.24 log CFU/g) compared to ST (5.38 log CFU/g) in the ceca at 7 dpi (*P* ≤ 0.05). Broilers from all 3 treatment groups had a similar *Salmonella* population in the ceca (approx. 3 log CFU/g) at 35 dpi. *Salmonella* concentrations in the liver, spleen and gizzard at 35 dpi were also similar for all 3 treatments with <1 log CFU/g (Table 3).

**Table 2:** *Salmonella* colonization and persistence (log CFU/g) in the ceca and internal organ invasion (liver and spleen) on d 7 of grow out as affected by different serovars (Typhimurium, Infantis, and Reading) and challenge dose (approx. 2, 5, and 8 log CFU/chick).

Part	Dose/p-values	*S*. Typhimurium	*S*. Infantis	*S*. Reading	Pooled results of 3 serovars
Ceca	Low	5.38 ± 0.79^x^	6.24 ± 0.71^y^	5.82 ± 0.41^a, xy^	6.65 ± 0.45
	Med	5.10 ± 0.62	6.09 ± 0.39	7.10 ± 0.46^c^	7.44 ± 0.27
	High	5.64 ± 0.47	6.39 ± 0.33	6.03 ± 0.52^b^	6.83 ± 0.22
	Linear	0.2972	0.2022	0.1468	0.2477
	Quad	0.4360	0.7596	0.0002	0.5278
Liver	Low	0.25 ± 0.32	1.14 ± 0.71	1.10 ± 0.72	1.6 ± 0.69
	Med	1.43 ± 0.95	1.92 ± 0.9	2.55 ± 1.13	2.93 ± 0.99
	High	0.89 ± 0.79	1.71 ± 0.81	2.14 ± 0.71	2.50 ± 0.90
	Linear	0.2730	0.4013	0.1232	0.0340
	Quad	0.0187	0.1238	0.0275	0.0344
Spleen	Low	0.49 ± 0.54^a^	1.33 ± 0.82	0.93 ± 0.64^a^	1.52 ± 0.79
	Med	1.19 ± 1.36^a^b	1.78 ± 0.31	2.26 ± 1.85^b^	3.12 ± 0.23
	High	1.33 ± 0.94b	2.05 ± 1.14	2.22 ± 0.88^b^	3.08 ± 0.59
	Linear	0.1718	0.1118	0.0807	0.0310
	Quad	0.7520	0.9501	0.3486	0.0932

Same superscripts (^abc^) within the same column indicate no significant differences (*P* > 0.05); same superscripts (^xy^) within the first 3 row (3 serovars of *S*. Typhimurium, *S*. Infantis, and *S*. Reading.) indicate no significant differences (*P* > 0.05).

**Table 3:** *Salmonella* colonization and persistence (log CFU/g) in the ceca and internal organ invasion (liver and spleen) on d 35 of grow out as affected by different serovars (Typhimurium, Infantis, and Reading) and challenge dose (approx. 2, 5 and 8 log CFU/chick).

Part	Dose/p-values	*S*. Typhimurium	*S*. Infantis	*S*. Reading	Pooled results of 3 serovars
Ceca	Low	1.67 ± 0.49^bx^	1.27 ± 0.7^bx^	3.02 ± 0.36^y^	3.28 ± 0.35
	Med	1.10 ± 1.1^abx^	0.19 ± 0.19^ax^	2.48 ± 2.48^y^	2.69 ± 0.85
	High	0.82 ± 0.82^bx^	0.25 ± 0.25^ax^	3.41 ± 3.41^y^	3.45 ± 0.71
	Linear	<0.001	<0.001	0.0026	0.6727
	Quad	0.7970	0.5074	0.1536	0.0626
Liver	Low	0.25 ± 0.33^x^	0.19 ± 0.19^x^	0.65 ± 0.52^y^	0.85 ± 0.76
	Med	0.08 ± 0.08^xy^	0 ± 0^x^	0.36 ± 0.36^y^	0.36 ± 0.34
	High	0 ± 0^x^	0 ± 0^x^	0.36 ± 0.36^y^	0.36 ± 0.22
	Linear	0.0706	0.0926	0.1401	0.1087
	Quad	0.0025	0.0157	0.0031	0.2334
Spleen	Low	0.04 ± 0.10	0.11 ± 0.2	0.13 ± 0.24	0.65 ± 0.73
	Med	0.14 ± 0.14	0.04 ± 0.04	0.31 ± 0.31	0.38 ± 0.29
	High	0.07 ± 0.07^xy^	0 ± 0^x^	0.29 ± 0.29^y^	0.39 ± 0.45
	Linear	0.048	0.0226	0.1016	0.3548
	Quad	0.0066	0.0125	0.0332	0.3640
Gizzard	Low	0.35 ± 0.44	0.51 ± 0.63^b^	0.18 ± 0.27	0.65 ± 0.73
	Med	0 ± 0	0 ± 0^a^	0.26 ± 0.26	0.26 ± 0.29
	High	0.09 ± 0.09	0.26 ± 0.26^ab^	0.09 ± 0.09	0.36 ± 0.45
	Linear	0.8234	0.155	0.3952	0.6319
	Quad	0.4778	0.8195	0.558	0.9724

Same superscripts (^abc^) within the same column indicate no significant differences (*P* > 0.05); same superscripts (^xy^) within the first 3 row (3 serovars of *S*. Typhimurium, *S*. Infantis, and *S*. Reading.) indicate no significant differences (*P* > 0.05).

### Persistence of *Salmonella* in Broiler Internal Organs

**Figure 1:**
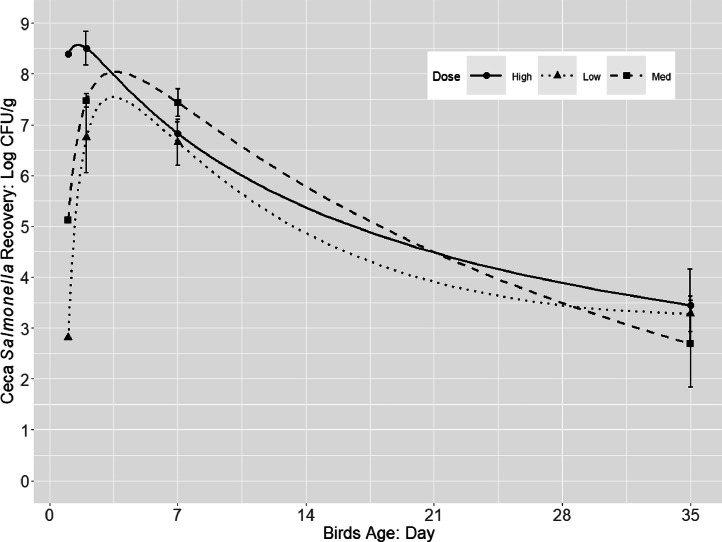
*Salmonella* population (log CFU/g; pooled counts of 3 serovars *S*. Typhimurium, *S*. Infantis, and *S*. Reading) in ceca of broilers during 35 d grow-out that were orally challenged with a 3 serovar cocktail of different challenge dose (approx. 2, 5 and 8 log CFU/chick).

*Salmonella* persisted in the ceca of the birds throughout the 35 d grow-out period, regardless of the challenge dose (Figure 1). *Salmonella* concertation in ceca decreased rapidly from 8.50 to 6.83 at 2 and 7 dpi, with a subsequent gradual decline to 3.45 log CFU/g by 35 dpi in H dose challenge group. A similar trend was observed for *Salmonella* concentration in the ceca of birds from L and M groups from ca. 7 log CFU/g at 2 dpi to ca. 3 log CFU/g at 35 dpi.

**Figure 2:**
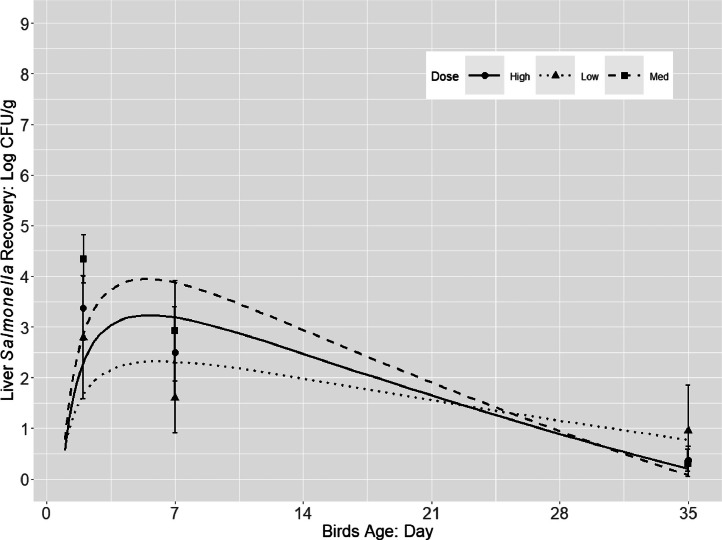
*Salmonella* population (log CFU/g; pooled counts of 3 serovars *S*. Typhimurium, *S*. Infantis, and *S*. Reading) in liver of broilers during 35 d grow-out that were orally challenged with a 3 serovar cocktail of different challenge dose (approx. 2, 5 and 8 log CFU/chick).

**Figure 3:**
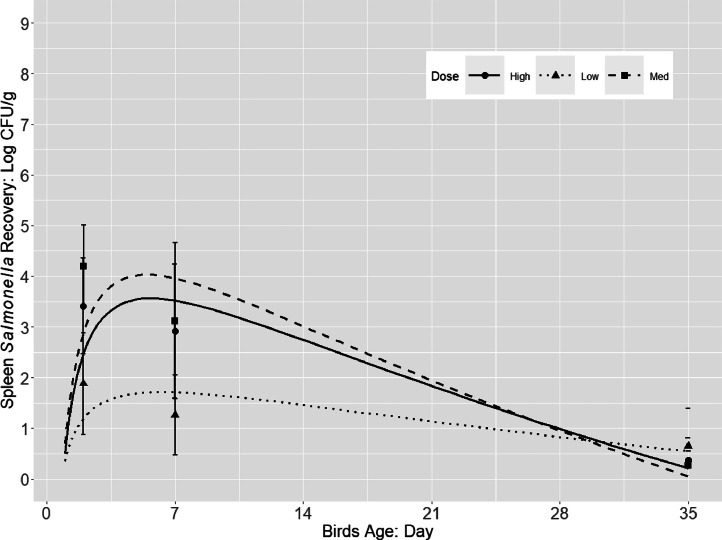
*Salmonella* population (log CFU/g; pooled counts of 3 serovars *S*. Typhimurium, *S*. Infantis, and *S*. Reading) in spleen of broilers during 35 d grow-out that were orally challenged with a 3 serovar cocktail of different challenge dose (approx. 2, 5 and 8 log CFU/chick).

Irrespective of the serovar or the challenge dose, *Salmonella* was able to colonize the ceca and invade the internal organs (spleen and liver). *Salmonella* populations increased quadratically in the liver and spleen during the early infection period of 2 and 7 dpi (approx. 2–4 log CFU/g) followed by a gradual decrease (<1 log CFU/g) up to 35 dpi (Figure 2, Figure 3). Different from *Salmonella* cecal colonization and persistence, M dose group resulted in higher *Salmonella* concentrations at 2 and 7 dpi (approx. 3–4 log CFU/g) compared to the other 2 doses (approx. 2–3 log CFU/g), although the concentration decayed over time to <1 log CFU/g for birds in all 3 treatment groups. Additionally, the difference of *Salmonella* population (log CFU/organ) in the spleen and liver at the same sampling time were less than 0.3 log CFU/g, which may suggest that the translocation is non-specific in broiler internal organs.

*Salmonella* population of all 3 serovars in broiler ceca and internal organs were similar (*P* > 0.05) at 2 and 7 dpi. However, SR showed greater persistence in the ceca (all 3 challenge dose groups) compared to ST and SI and numerically higher counts in the liver, spleen and gizzard at 35 dpi.

### Logistic Regression

**Figure 4:**
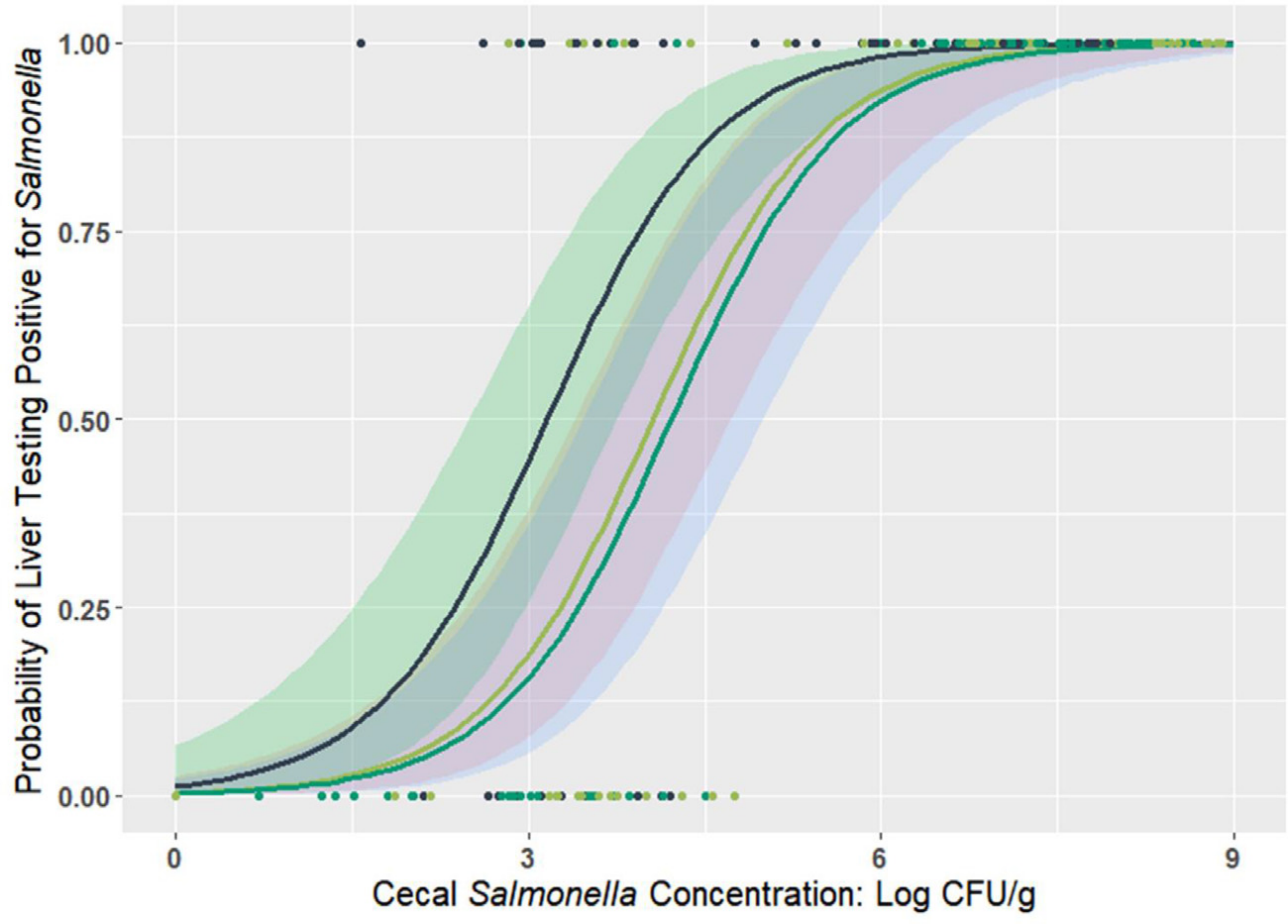
Probability of liver *Salmonella* positivity in broilers that were orally challenged with a 3-serovar cocktail of *Salmonella* (*S*. Typhimurium, *S*. Infantis, and *S*. Reading) of different challenge dose (ca. 2, 5 and 8 log CFU/chick).

**Figure 5:**
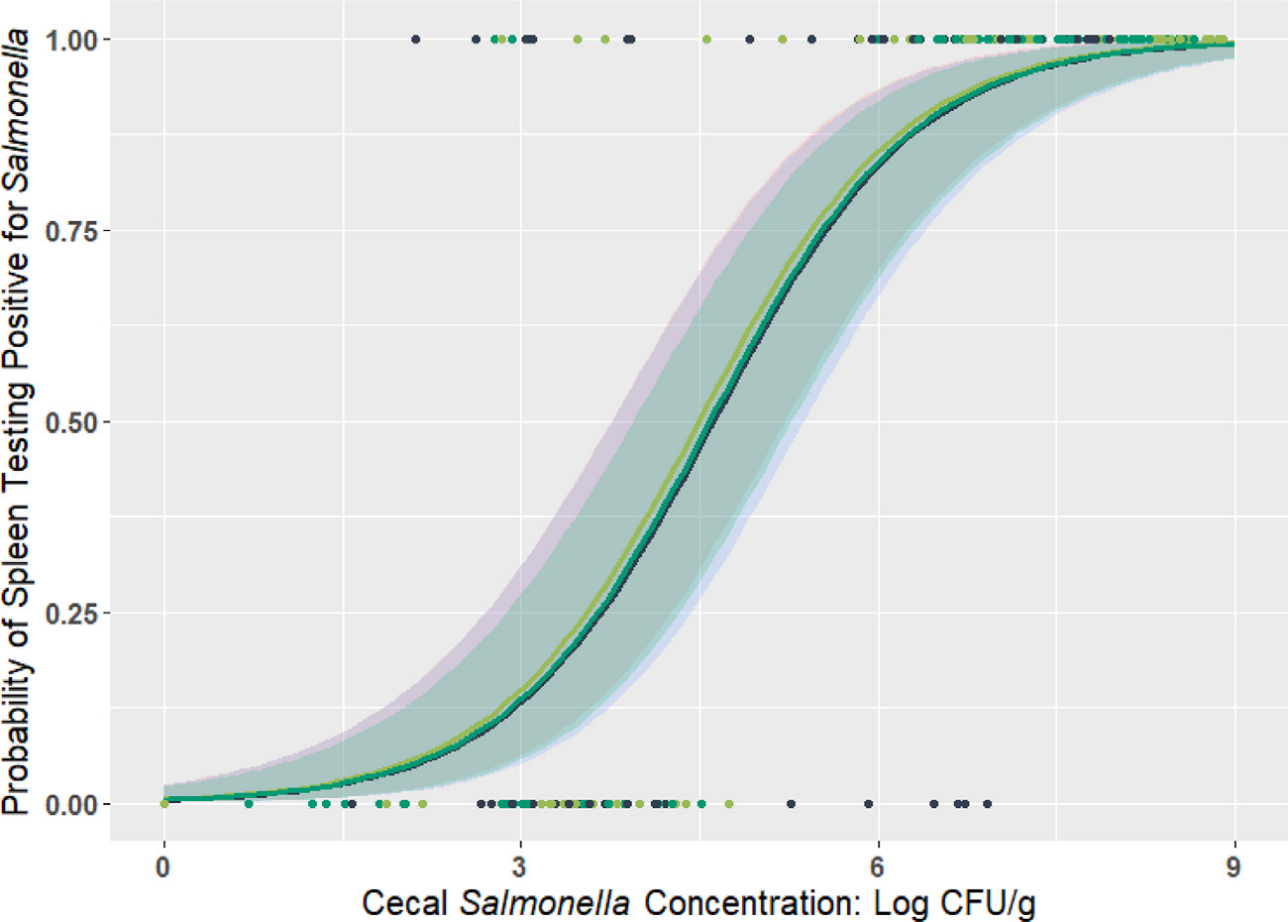
Probability of spleen *Salmonella* positivity in broilers that were orally challenged with a 3-serovar cocktail of *Salmonella* (*S*. Typhimurium, *S*. Infantis, and *S*. Reading) of different challenge dose (ca. 2, 5 and 8 log CFU/chick).

Results of logistic regression were presented in Figure 4, Figure 5. The estimates and statistics of the logistic regression model is shown in Table 4. As the increase of cecal *Salmonella* concentration from 0 to 9 log CFU/g, the probability of liver or spleen to be tested positive for *Salmonella* increased from 0 to almost 100%. More specifically, for every 1-log CFU/g increase in cecal *Salmonella* load, the relative risk of liver and spleen being *Salmonella* positive increased by 4.02 and 3.40 times. Moreover, broilers from H and M group had a lower risk (28 and 23%, *P*≤0.05) of liver being positive for *Salmonella* compared to the birds in L group for the same cecal concentration.

**Table 4:** Correlation of cecal *Salmonella* population and *Salmonella*-positivity of liver and spleen in broilers challenged with various doses of *Salmonella* (ca. 2, 5, and 8 log CFU/chick) over 35 d period.

Organs	Liver			Spleen		
terms	estimate	Odds ratio/ relative risk	*P* value	Estimate	Odds ratio/ relative risk	*P* value
Intercept	−4.3938	0.01	<0.001	−5.3852	0.01	<0.001
Ceca	1.3907	4.02	<0.001	1.1657	3.21	<0.001
Med	−1.4685	0.23	0.030	0.0387	1.04	0.949
High	−1.2517	0.29	0.045	0.1448	1.16	0.799

## DISCUSSION

Oral and/or cloacal challenges were the more common and effective routes for colonization of *Salmonella* in the gastrointestinal tract, with a faster colonization and invasion of the internal organs achieved via the cloacal route (Bailey et al., 2005). We used the oral challenge as it replicates the normal oral-fecal route of transmission of the chicks in a poultry house, either through the feed, water, litter, pests, or other sources in poultry production and also to achieve a consistent colonization of the 1-day-old chicks.

Oral challenge of *Salmonella* (7–9 log CFU/bird) in young broilers has been commonly used to establish the infection model that is used to evaluate nutritional strategies on controlling *Salmonella* in broiler live production (Knap et al., 2011; Shanmugasundaram et al., 2019). The authors reported that *Salmonella* challenge resulted in colonization of the chicks followed by translocation to other body tissues and persisted throughout the 5-wk grow-out period in the current study. The low pH of the upper gastrointestinal tract is a contributing factor for the higher level of *Salmonella* required for colonizing young chicks (Bailey et al., 2005). However, even the L dose challenge (ca. 2 log CFU/bird) resulted in successful colonization and invasion of the liver and spleen in the present study, probably due to the immature gastrointestinal tract of newly hatched chicks. A higher *Salmonella* challenge dose in broilers resulted in a linear increase in the cecal colonization (Bailey et al., 2005). Additionally, a challenge dose of 2-8 log CFU/bird of *Salmonella* resulted in a high population of *Salmonella* (6-8 log CFU/g) in the ceca during the first week. reported that a challenge dose of 3 log CFU/bird of *S.* Typhimurium in 1-day-old chicks resulted in a 5 log CFU/g in ceca and ileum by d 8 dpi. *Salmonella* concentration decreased gradually during the grow-out, with ca. 3 log CFU/g *Salmonella* recovered from cecal samples at 35 dpi. Stern (2008) reported cecal *Salmonella* concentration decayed rapidly from 6 to 1 log CFU/g during 28 d period in birds challenged from 4-8 log CFU/bird of *Salmonella*.

During *in vivo Salmonella* challenge trials, regardless of oral challenge or aerosol exposure, *Salmonella* cecal population decreased gradually over time after the challenge (Marcq et al., 2011; Pal et al., 2021). Bjerrum et al. (2003) also reported a rapid decrease in *Salmonella* population in the ileum and ceca following a challenge with 7.2 log CFU of *S.* Typhimurium on day of hatch, over a 35-d period in isolators. With the natural coprophagic behavior, raising broilers in floor pens with access to contaminated litter may lead to continuous colonization of the gastrointestinal tract and subsequent spread to their internal organs. Yadav et al. (2022) reported similar *Salmonella* concentration in broiler ceca was <1 log CFU/g subsequent to 21 d of grow-out when the chicks/birds were grown in battery cages (limiting re-exposure to *Salmonella* from the environmental sources), using the same ST serovar and challenged with 7 log CFU/bird on d 1. However, Bohorquez (2007) reported that broilers reared in cages or on litter had a higher concentration of *Salmonella* in the ceca (ca. 3 log CFU/g) on d 42, in chicks orally challenged on d 3 with non-marked strains of *Salmonella* serovars. The differences of *Salmonella* colonization in the ceca of birds reared in cages and floor pens may be due to the marker strains used for challenge or differences in the serovars/strains of *Salmonella* used for the challenge. The use of marked strains minimizes the impact of background microbiota in the chicken gut on enumeration and allows a more precise tracking of *Salmonella* population (Bailey et al., 2005). The findings of the present study also suggest that when resources allow, *Salmonella* challenge of 1-day-old chicks followed by rearing on floor pens may be a more reliable model for evaluating the effectiveness of dietary interventions during broiler production/grow-out. This represents real life scenario, where the 1-day-old chicks are exposed to *Salmonella* in the hatchery as well as in the poultry house environment after placement. Meanwhile, an oral *Salmonella* challenge concentration at 2 to 5 log CFU/bird is adequate to colonize the 1-day-old chicks under those rearing conditions.

In addition to the gradual decrease in *Salmonella* population in the ceca, broilers challenged with 3 doses eventually stabilize at comparable *Salmonella* concentration in the ceca (ca. 3 log CFU/g) at 35 d of age. Under commercial production, exposure of 1-day-old chicks to *Salmonella* is probably limited at a low or medium *Salmonella* concentrations as the litter has been reported to contain low levels of *Salmonella*, with a mean population of 1.70 log MPN/g (range of <1.0 to 3.6 log MPN/g; Gutierrez et al., 2020). Regardless of the *Salmonella* concentration in the environmental sources such as water, feed or litter, *Salmonella* exposure can result in exponential population increase in in the GI tract, followed by persistent colonization in the ceca and invasion of internal organs (spleen and liver). It is possible that the birds entering the processing plant may carry up to 3-log of *Salmonella* populations in the ceca and antimicrobial interventions designed at processing facilities should be able to address and reduce these *Salmonella* populations (resulting from cross contamination of the carcass during processing) to assure the safety of poultry meat.

Despite several limitations in the logistic regression currently used such as that data points were acquired from a mixture of 2, 7, and 35 d-old birds, and the cecal concentrations were only at ca. 3 log CFU/g at 35 d of age. The current results suggested that a higher concentration of *Salmonella* in ceca is associated with a higher probability that other internal organs (liver and spleen) tare positive for *Salmonella*. However, a lower risk was observed in birds challenged with H and M doses of *Salmonella*. A possible explanation is that the immune system plays a role in controlling *Salmonella* translocation. Previous studies have shown that when a *Salmonella* challenge was subclinical and asymptotic, intestinal integrity was not affected after 6 d post inoculation (Yadav et al., 2022). Gut permeability increased 72 h after broilers were challenged with *Salmonella* Enteritidis (Prado-Rebolledo et al., 2017). Choi et al. (2022) reported broilers challenged with *Salmonella* exhibited gut leakage at 14 d but recovered by 21 d of age. The differences in gut permeability suggests that intestinal immune regulation is related to the infection time as well as the maturation of the immune system. Additional research is necessary to elucidate immune response of *Salmonella* challenged birds and possibly use it as an intervention method to reduce *Salmonella* incidence in broilers prior to harvest. The findings of the present study indicate a possible link between the *Salmonella* concentration at pre-harvest (in litter, cecal droppings, or cecal contents) and its prevalence in other internal organs, necessitating further investigation. Spread of *Salmonella* to internal organs (liver and spleen) requires crossing of the microorganisms through the GI tract and subsequent systemic translocation and colonization (Adhikari et al., 2020; Shi et al., 2021; Choi et al., 2022). *Salmonella* translocation to internal organs such as the liver and spleen is often reported as prevalence, rather than the population. Microbial risk assessments for the safety of chicken parts such as liver, gizzard and others consumed as food require quantitative data to evaluate the risk and identify risk reduction strategies. Data on the *Salmonella* concentration in liver and spleen in market age birds (broilers) are lacking. Higher *Salmonella* challenge dose resulted in a linear increase in cecal *Salmonella* population. However, the concentration of *Salmonella* recovered from liver and spleen quadratically increased with increasing challenge dose and stabilized at M dose group. The differences in the *Salmonella* concentrations observed in the ceca versus the liver and spleen may be due to the immune response of the bird. Immunity to *Salmonella* infection in young chicks is mainly from the maternal antibodies and the activity of both innate and adaptive immune system, with natural killer cells, intraepithelial T cells and macrophages as critical factors (Meijerink et al., 2021). Spleen, as the secondary immune organ not only functions as blood filtration, but also is a key immune organ for systemic antigen sampling and mounting appropriate humoral and/or cell-mediated immune responses (Smith and Hunt, 2004). Hamad et al. (2017) reported that host-defense peptides play a key role in the innate immunity components with direct antimicrobial activities and immunomodulatory properties in poultry species. Exposing the chicks to a high *Salmonella* population (8 log CFU/g) as in the H dose challenge possibly could trigger a more acute immune response and upregulation of related immune genes such as avian beta-defensins and cathelicidins. This results in fewer *Salmonella* cells disseminated systematically to the liver and spleen in comparison to the lower challenge doses (L and M). Additional studies are necessary to evaluate the relationship between the challenge dose and the immune responses of the birds.

Foodborne outbreaks and illnesses from consumption of inadequately cooked, contaminated chicken livers and gizzards have been reported over the past 20 yr (Abdalrahman and Fakhr, 2015; Lanier et al., 2018). Jung et al. (2019) reported an overall *Salmonella* prevalence of 59.4% with concentration from 6.4 to 254 MPN/g on chicken liver products in the U.S. Oscar (2021) reported a median *Salmonella* concentration of 1.8 log MPN/58 g (average weight of one chicken liver) at a retailer in Maryland. The *Salmonella* detected in products in the market may be due to the microorganisms present in the product (inside tissues) resulting from colonization of the GI tract and subsequent spread to other tissues (e.g., liver) or on the surface resulting from cross contamination during processing (Byrd et al., 2002; Berrang et al., 2019). In the present study, *Salmonella* concentrations in the liver, spleen, and gizzard decreased to <1 log CFU/g in market age birds (35 d). Irrespective of the challenge dose, all 3 *Salmonella* serovars were able to colonize the ceca and cause subsequent systemic translocation to other tissues (liver and liver) as well as in the gizzard at market age. Nagel et al. (2013) stated that interventions at pre-harvest stage that can reduce *Salmonella* populations to ca. 2-log in the ceca could successfully reduce the risk of *Salmonella* in edible parts (e.g., gizzard and liver) and consequent risk of foodborne illness from consumption of these parts. The results from the current study corroborated that *Salmonella* in the chicken gut can translocate and persist in broiler internal organs during live production. Therefore, incorporating effective antimicrobial interventions on those edible organs to address both the internal as external (surface) *Salmonella* contamination becomes necessary at the post-harvest stage to minimize the *Salmonella* contamination.

*Salmonella* serotypes Typhimurium and Infantis were linked to foodborne illness outbreaks and illnesses from consumption of chicken meat, while *S.* Reading was recognized as a significant issue with turkey products including ground meat and burgers (Hassan et al., 2019; CDC, 2019). Tyson et al. (2021) reported rapid spread of an emergent *Salmonella* Infantis clone with a large megaplasmid (**pESI**) containing the extended-spectrum beta-lactamase gene, which may be associated to the sharp increase in the illness linked to Infantis recently. Results from the present study illustrated that *S*. Reading can colonize chicken gut and could be a potential threat to broiler production as well, being a dominant strain over *S.* Typhimurium and *S.* Infantis, since it persisted in broiler ceca at 35 d. Additionally, *S*. Reading spread to the internal organs (liver, spleen, and gizzard) was observed, although foodborne illness outbreaks of *S*. Reading have not been linked to chicken meat. It is interesting that a higher population *S*. Reading was observed in the ceca at 35 d compared to Typhimurium and Infantis. There is a need for further research to evaluate this comparative advantage of these serovars in the chicken gut. Measures to minimize the sources of *Salmonella* for the birds and subsequent colonization and spread to other organs as well as measures to reduce the *Salmonella* populations in the birds at market age should be implemented at pre-harvest stage during broiler production.

## CONCLUSIONS

A low challenge dose of *Salmonella* (2 log CFU/bird) was adequate to colonize the GI tract of 1-day old chicks and subsequent spread to internal organs. Regardless of the *Salmonella* challenge dose and the concentrations subsequent to the challenge, similar populations of *Salmonella* were observed in the liver, spleen, and gizzard at market age (35 d) of these broilers. *Salmonella* Reading showed a greater persistence and population in the chicken ceca at market age, although the populations in the liver, spleen and gizzard were similar to other serovars (Typhimurium and Infantis). Meanwhile, reducing the cecal *Salmonella* load reduced the prevalence in liver and spleen. The *Salmonella* populations in chicken organ meats (liver and gizzard) at market age (35 d) were low, and conventional cooking practices used for preparation of these chicken parts should be adequate to eliminate the risk of bacterial survival and minimize occurrence of foodborne illness outbreaks.

## References

[bib0001] Abdalrahman L.S., Fakhr M.K. (2015). Incidence, antimicrobial susceptibility, and toxin genes possession screening of *Staphylococcus aureus* in retail chicken livers and gizzards. Foods..

[bib0002] Adhikari P., Yadav S., Cosby D.E., Cox N.A., Jendza J.A., Kim W.K. (2020). Research Note: Effect of organic acid mixture on growth performance and *Salmonella* Typhimurium colonization in broiler chickens. Poult. Sci..

[bib0003] Alba P., Leekitcharoenphon P., Carfora V., Amoruso R., Cordaro G., Di Matteo P., Ianzano A., Iurescia M., Diaconu E.L., Study Group E.N., Pedersen S.K., Guerra B., Hendriksen R.S., Franco A., Battisti A. (2020). Molecular epidemiology of *Salmonella* Infantis in Europe: insights into the success of the bacterial host and its parasitic pESI-like megaplasmid. Microb. Genom..

[bib0004] Antunes P., Mourão J., Campos J., Peixe L. (2016). Salmonellosis: the role of poultry meat. Clin. Microbiol. Infect.

[bib0005] Bailey J.S., Cox N.A., Cosby D.E., Richardson L.J. (2005). Movement and persistence of *Salmonella* in broiler chickens following oral or intracloacal inoculation. J. Food Prot..

[bib0006] Berrang M.E., Meinersmann R.J., Cox N.A. (2019). *Campylobacter* subtypes detected in broiler ceca and livers collected at slaughter1. Poult. Sci..

[bib0007] Bjerrum L., Engberg R.M., Pedersen K. (2003). Infection models for *Salmonella* Typhimurium DT110 in day-old and 14-day-old broiler chickens kept in isolators. Avian Dis.

[bib0008] Bohorquez D.V. (2007). Dietary and Housing Effects on Growth Performance, Gut Health and *Salmonella* Colonization of *Salmonella*-Challenged Broilers.

[bib0009] Byrd J.A., Hargis B.M., Corrier D.E., Brewer R.L., Caldwell D.J., Bailey R.H., McReynolds J.L., Herron K.L., Stanker L.H. (2002). Fluorescent marker for the detection of crop and upper gastrointestinal leakage in poultry processing plants. Poult. Sci..

[bib0010] Centers for Disease Control and Prevention (2019).

[bib0013] Cobb-Vantress, 2018a. Broiler performance and nutrition. Supplement.

[bib0014] Cobb-Vantress, 2018b. Cobb Broiler Management Guide.

[bib0012] Choi J., Marshall B., Ko H., Shi H., Singh A.K., Thippareddi H., Holladay S., Gogal R.M., Kim W.K. (2022). Antimicrobial and immunomodulatory effects of tannic acid supplementation in broilers infected with *Salmonella* Typhimurium. Poult. Sci..

[bib0016] European Food Safety Authority (2018). The European Union summary report on trends and sources of zoonoses, zoonotic agents and foodborne outbreaks in 2017. Eur. Food Safety Authority J.

[bib0017] Finstad S., O'Bryan C.A., Marcy J.A., Crandall P.G., Ricke S.C. (2012). *Salmonella* and broiler processing in the United States: relationship to foodborne salmonellosis. Food Res. Int..

[bib0019] Gutierrez A., De J., Schneider K.R. (2020). Prevalence, concentration, and antimicrobial resistance profiles of *Salmonella* isolated from Florida poultry litter. J. Food Prot..

[bib0020] Hamad S.K., Kim S., El-Kadi S.W., Wong E.A., Dalloul R.A. (2017). Comparative expression of host defense peptides in turkey poults. Poult. Sci..

[bib0021] Harris L., Farber J.N., Beuchat L.R., Parish M.E., Suslow T.V., Garrett E.H., Busta F.F. (2003). Outbreaks associated with fresh produce: incidence, growth, and survival of pathogens in fresh and fresh-cut produce. Comp. Rev. Food Sci. Food Safety.

[bib0022] Hassan R., Buuck S., Noveroske D., Medus C., Sorenson A., Laurent J., Rotstein D., Schlater L., Freiman J., Douris A., Simmons M., Donovan D., Henderson J., Tewell M., Snyder K., Oni O., Von Stein D., Dassie K., Leeper M., Adediran A., Dowell N., Gieraltowski L., Basler C. (2019). Multistate outbreak of *Salmonella* infections linked to raw turkey products - United States, 2017-2019. Morbidity Mortality Weekly Rep..

[bib0023] Interagency Food Safety Analytics Collaboration (2021).

[bib0024] Jung Y., Porto-Fett A.C., Shoyer B.A., Henry E., Shane L.E., Osoria M., Luchansky J.B. (2019). Prevalence, levels, and viability of *Salmonella* in and on raw chicken livers. J. Food Prot..

[bib0025] Knap I., Kehlet A.B., Bennedsen M., Mathis G.F., Hofacre C.L., Lumpkins B.S., Jensen M.M., Raun M., Lay A. (2011). Bacillus subtilis (DSM17299) significantly reduces *Salmonella* in broilers. Poult Sci.

[bib0026] Lamas A., Miranda J.M., Regal P., Vázquez B., Franco C.M., Cepeda A. (2018). A comprehensive review of non-enterica subspecies of *Salmonella enterica*. Microbiol. Res..

[bib0027] Lanier W.A., Hale K.R., Geissler A.L., Dewey-Mattia D. (2018). Chicken liver–associated outbreaks of campylobacteriosis and salmonellosis, United States, 2000–2016: Identifying opportunities for prevention. Foodborne Pathog. Dis..

[bib0028] Marcq C., Cox E., Szalo I.M., Théwis A., Beckers Y. (2011). *Salmonella* Typhimurium oral challenge model in mature broilers: bacteriological, immunological, and growth performance aspects. Poult. Sci.

[bib0029] Meijerink N., van den Biggelaar R.H.G.A., van Haarlem D.A., Stegeman J.A., Rutten V.P.M.G., Jansen C.A. (2021). A detailed analysis of innate and adaptive immune responsiveness upon infection with *Salmonella enterica* serotype Enteritidis in young broiler chickens. Vet. Res..

[bib0030] Nagel G.M., Bauermeister L.J., Bratcher C.L., Singh M., McKee S.R. (2013). *Salmonella* and *Campylobacter* reduction and quality characteristics of poultry carcasses treated with various antimicrobials in a post-chill immersion tank. Int. J. Food Microbiol..

[bib0031] Oscar T.P. (2021). Monte Carlo simulation model for predicting *Salmonella* contamination of chicken liver as a function of serving size for use in quantitative microbial risk assessment. J. Food Prot..

[bib0032] Pal A., Riggs M.R., Urrutia A., Osborne R.C., Jackson A.P., Bailey M.A., Macklin K.S., Price S.B., Buhr R.J., Bourassa D.V. (2021). Investigation of the potential of aerosolized *Salmonella* Enteritidis on colonization and persistence in broilers from day 3 to 21. Poult. Sci..

[bib0033] Prado-Rebolledo O.F., Delgado-Machuca J.D.J., Macedo-Barragan R.J., Garcia-Márquez L.J., Morales-Barrera J.E., Latorre J.D., Hernandez-Velasco X., Tellez G. (2017). Evaluation of a selected lactic acid bacteria-based probiotic on *Salmonella enterica* serovar Enteritidis colonization and intestinal permeability in broiler chickens. Avian Pathol.

[bib0015] Punchihewage-Don A.J., Hawkins J., Adnan A.M., Hashem F., Parveen S. (2022). The Outbreaks and Prevalence of Antimicrobial Resistant *Salmonella* in Poultry in the United States: an overview. Heliyon.

[bib0034] R Core Team (2021).

[bib0035] Shanmugasundaram R., Mortada M., Cosby D.E., Singh M., Applegate T.J., Syed B., Pender C.M., Curry S., Murugesan G.R., Selvaraj R.K. (2019). Synbiotic supplementation to decrease *Salmonella* colonization in the intestine and carcass contamination in broiler birds. PloS one.

[bib0036] Shi H., Deng X., Deng Q., Liu Z., Liu N. (2021). Probiotic lactobacilli improved growth performance and attenuated *Salmonella* Typhimurium infection via jak/stat signaling in broilers. Braz. J. Poult. Sci..

[bib0037] Siceloff A.T., Waltman D., Shariat N.W., Elkins C.A. (2022). Regional *Salmonella* differences in United States broiler production from 2016 to 2020 and the contribution of multiserovar populations to *Salmonella* surveillance. Appl. Environ. Microbiol..

[bib0038] Smith K.G., Hunt J.L. (2004). On the use of spleen mass as a measure of avian immune system strength. Oecologia.

[bib43] Stern N.J. (2008). *Salmonella* species and *Campylobacter jejuni* cecal colonization model in broilers. Poult. Sci..

[bib0039] Tack, D. M., L. Ray, P. M. Griffin, P. R. Cieslak, J. Dunn, T. Rissman, R. Jervis, S. Lathrop, A. Muse, M. J. M. Duwell, and M. W. Report. 2020. Preliminary incidence and trends of infections with pathogens transmitted commonly through food- Foodborne Diseases Active Surveillance Network, 10 US Sites, 2016–2019. 69:509. 10.15585/mmwr.mm6917a1 PMC7206985 32352955

[bib0040] Tyson G.H., Li C., Harrison L.B., Martin G., Hsu C.H., Tate H., Tran T.T., Strain E., Zhao S. (2021). A multidrug-resistant *Salmonella* Infantis clone is spreading and recombining in the United States. Microbial Drug Resistance.

[bib0041] Yadav S., Teng P.Y., Choi J., Singh A.K., Vaddu S., Thippareddi H., Kim W.K. (2022). Influence of rapeseed, canola meal and glucosinolate metabolite (AITC) as potential antimicrobials: Effects on growth performance, and gut health in *Salmonella* Typhimurium challenged broiler chickens. Poult Sci.

